# Beet curly top virus affects vector biology: the first transcriptome analysis of the beet leafhopper

**DOI:** 10.1099/jgv.0.002012

**Published:** 2024-07-29

**Authors:** Jinlong Han, Meihua Cui, Jordan Withycombe, Max Schmidtbauer, Judith Chiginsky, Oliver T. Neher, Carl A. Strausbaugh, Rajtilak Majumdar, Vamsi J. Nalam, Punya Nachappa

**Affiliations:** 1 Department of Agricultural Biology, Colorado State University, Fort Collins, CO, USA; 2 Amalgamated Sugar Company, Boise, ID, USA; 3 USDA-ARS NWISRL, 3793 North 3600 East, Kimberly, ID, USA

**Keywords:** *Curtovirus*, *Circulifer tenellus*, fecundity, transcriptome, virus-vector interaction

## Abstract

Curly top disease, caused by beet curly top virus (BCTV), is among the most serious viral diseases affecting sugar beets in western USA. The virus is exclusively transmitted by the beet leafhopper (BLH, *Circulifer tenellus*) in a circulative and non-propagative manner. Despite the growing knowledge on virus-vector interactions, our understanding of the molecular interactions between BCTV and BLH is hampered by limited information regarding the virus impact on the vector and the lack of genomic and transcriptomic resources for BLH. This study unveils the significant impact of BCTV on both the performance and transcriptome response of BLHs. Viruliferous BLHs had higher fecundity than non-viruliferous counterparts, which was evident by upregulation of differentially expressed transcripts (DETs) associated with development, viability and fertility of germline and embryos in viruliferous insects. Conversely, most DETs associated with muscle movement and locomotor activities were downregulated in viruliferous insects, implying potential behavioural modifications by BCTV. Additionally, a great proportion of DETs related to innate immunity and detoxification were upregulated in viruliferous insects. Viral infection also induced notable alterations in primary metabolisms, including energy metabolism, namely glucosidases, lipid digestion and transport, and protein degradation, along with other cellular functions, particularly in chromatin remodelling and DNA repair. This study represents the first comprehensive transcriptome analysis for BLH. The presented findings provide new insights into the multifaceted effects of viral infection on various biological processes in BLH, offering a foundation for future investigations into the complex virus-vector relationship and potential management strategies for curly top disease.

## Introduction

Curly top disease, caused by beet curly top virus (BCTV, family: *Geminiviridae*, genus: *Curtovirus*), is one of the most economically important diseases, which can infect over 300 plant species, including key crops like common bean, cucurbits, hemp, peppers, tomatoes and sugar beets in the western USA [1,3]. The virus is characterized by a positive-sense, single-stranded circular DNA genome, encapsulated within twinned quasi-isometric virions [4]. There are 11 different BCTV strains, which vary over time by geographic regions and can often be observed as mixed infections [5]. BCTV is a phloem-limited virus that is exclusively transmitted by the beet leafhopper (BLH), *Circulifer tenellus* (Hemiptera: Cicadellidae). The epidemiology of BCTV is dependent on the migration patterns of its insect vector. BLHs can be found throughout the semi-arid regions of the western and southwestern USA, and their migration patterns are influenced by numerous factors, including climate, feeding preference, food availability and leafhopper reproduction [6]. BLHs are highly polyphagous and reproduce on a variety of crops and weed species, making them prolific vectors of BCTV [2]. BLHs overwinter as adult females on perennial hosts or winter annuals [2]. Each female can lay around 200–300 eggs, which are deposited into leaf and stem tissues in the spring. Eggs hatch in 5–40 days and mature over five nymphal instars. The life cycle and number of generations per year are dependent on temperature, rainfall, availability of hosts and other factors but can be as many as three to five or more generations per growing season in the USA (reviewed in [7]).

The mode of virus transmission is circulative and non-propagative, where the virus circulates through the insect’s body without replication [2,7]. Virus acquisition by BLHs can occur in as little as 1–2 min of feeding on an infected host plant, with extended feeding periods (e.g. acquisition access period of 48 h) leading to a higher efficiency in virus transmission [8]. There is a 4 h latent period for the virions to migrate throughout the insect gut and hemolymph and then into the salivary glands before transmission. Adult insects can retain and spread the virus during their entire 1–2-month lifespan [9,10]. The BLH can acquire enough virions to maintain lifelong transmission potential, but the transmission rate and the amount of virus in the insect decrease over time if not maintained on a susceptible host, which suggests that the virus does not replicate in the insect [7]. Virus transmission efficiency is also influenced by the insect life stage and sex [11,12]. One study reported smaller instar nymphs to be more efficient transmitters than larger ones [11], while another study identified third instar nymphs as the most effective [12]. This inconsistency underscores the need for further investigation into the stage-dependent differences in virus transmission. Adult males surpass females in transmission efficiency, but there is no difference between male and female nymphs. Additionally, young adult leafhoppers are more efficient virus vectors than older adults [11].

There is ample evidence of vector manipulations by plant viruses that alter vector behaviour, physiology and virus transmission. These effects can occur indirectly through plant-mediated mechanisms or directly within the vector following virus acquisition (reviewed in [13,14]). Several studies have demonstrated the occurrence of vector manipulations by plant viruses across various pathosystems involving aphids [15,18], whiteflies [19] and thrips [20,23]. To date, there are only two published reports of BCTV manipulation of its vector. One study showed a prolonged BLH nymphal development on BCTV-infected plants compared to healthy plants, with no observable impact on longevity and fecundity [24]. More recently, Lee and colleagues (2022) demonstrated that non-viruliferous BLHs preferred to probe on barley (*Hordeum vulgare*, BCTV non-host) and ribwort plantain (*Plantago lanceolata*, BCTV host) over tomato (*Solanum lycopersicum*, BCTV host), while viruliferous insects showed no preference. This change in probing preference in viruliferous BLHs may result in enhanced BCTV transmission in tomato fields [25]. Despite these insights into BCTV-vector interactions, the lack of genomic and transcriptomic resources for this crucial vector has limited our understanding of virus-vector interactions and hindered our capacity to develop novel strategies for vector control. In this study, we assessed the impact of BCTV on vector performance and conducted RNA sequencing (RNA-Seq) to explore the underlying transcriptomic responses of BLH to BCTV acquisition.

## Methods

### Insect and virus source

Beet curly top virus-infected (viruliferous) BLH were originally collected in southern Idaho and shipped to Colorado State University in Fort Collins, CO. To obtain non-viruliferous insects, eggs hatching from plant tissues were observed under a dissecting microscope, and the first instar nymphs were immediately removed from the leaf tissues with a fine paintbrush and moved to a BCTV-free sugar beet plant (cultivar BPA9000). Both viruliferous and non-viruliferous colonies were maintained on sugar beet plants at a temperature of 22–24 °C under a 16L:8D photoperiod in insect cages (30.48 m × 30.48 m × 30.48 m, BioQuip Products Inc., Rancho Dominguez, CA) in separate growth chambers. The colony plants were cultivated in circular pots (12.7 cm × 12.7 cm) and fertilized on a bi-monthly basis with Osmocote 15-9-12 N:P:K time release fertiliser (Scott’s Company, Marysville, OH). The virus status of the colonies was tested monthly by PCR. Briefly, DNA was extracted from ten individual adult leafhoppers and colony plants using the Wizard Genomic DNA Purification Kit (Promega) following the manufacturer’s instructions. PCR was conducted using the BCTV-specific primer pair designed by Strausbaugh et al. 2017 [5] (Table S1, available in the online Supplementary Material) under the following thermocycling conditions: initial denature of 95 °C for 5 min, followed by 40 cycles of 95 °C for 1 min, 58 °C for 1 min and 72 °C for 1 min, and a final extension step of 72 °C for 5 min.

### Insect performance assay

The performance of viruliferous and non-viruliferous BLHs was assessed in a growth chamber using sugar beet plants at the four–six leaf stage (6 weeks old). To initiate the assay, viruliferous or non-viruliferous late instar (three–five stage) nymphs were obtained from the lab colony and maintained in an insect cage until adult eclosion and sexed. A total of six even-aged adult leafhopper, four females and two males, were placed on a single uninfected sugar beet plant, and females were given a 7-day oviposition period and then removed. Observations were recorded every other day for the emergence of nymphs (fecundity), nymph development time, nymph survival and number of adults. There were 39 biological replicates (individual plant/cage) for viruliferous insects and 27 replicates for non-viruliferous insects.

### RNA extraction, library preparation and RNA sequencing

Total RNA was extracted from a pool of ~50 even-aged viruliferous or non-viruliferous adult BLHs (40 females and 10 males) using RNeasy Mini Qiagen kit (Qiagen Inc., Valencia, CA). Three independent biological replicates per treatment were included in the RNA-Seq experiment. RNA yields and qualities were evaluated using Nanodrop One Spectrophotometer (Thermo Scientific, Waltham, MA) and Agilent 2100 Bioanalyzer system (Agilent Technologies, Santa Clara, CA). Subsamples of total RNA isolated from insect tissue were treated with DNase using the rigorous DNA removal procedure of the Turbo DNA-free kit (Applied Biosystems Inc, Carlsbad, USA) prior to submission to the CSU Genomics Core for cDNA library preparation and sequencing. The cDNA libraries were constructed using NEBNext Ultra II Directional RNA library prep kit (Illumina, San Diego, CA, USA) and subjected to paired-end (2×150 bp) sequencing using Illumina Hi-Seq 2000 platform. The raw sequencing reads have been deposited in the NCBI SRA database under the BioProject accession PRJNA1089802.

### Genome-guided transcriptome assembly

Raw reads were trimmed for adapter sequences using Fastp v0.23.2 [26]. Subsequently, high-quality reads were obtained by removing reads with Phred score less than 25 and length shorter than 100 bp. After trimming, an average of 97 and 91 % of reads across the libraries had Phred scores higher than 20 and 30, respectively. A statistical summary of trimmed reads is provided in Table S2. The paired-end reads were mapped to the CTen_1 reference genome assembly (GenBank assembly GCA_030545055.1 submitted as BioProject PRJNA933715 by the USDA-ARS sugar beet research group in Kimberly, ID) using Hisat2 v2.2.1 with the default parameters. The mapped sequences were further assembled into transcripts using Stringtie v2.2.1 with the default values [27]. The quality and completeness of the assembly were evaluated using Transrate v2.14.0 [28] and Benchmarking Universal Single-Copy Orthologs (BUSCOs) v5.2.2 [29] against the lineage dataset of Arthropoda Odb10 (Table 1 and Fig. S1).

**Table 1.** Statistic summary of genome-guided transcriptome assembly for the BLH

Measurement*	No.
Total assembled contigs/transcripts	63 534
Total assembled bases	125 777 099
Mean length of contigs (bp)	1979
N50 of contigs	3160
Min/Max contig length (bp)	66/59 294
Sequences with open reading frame	48 224
Read mapping rate to genome reference	89 %
BUSCO score for complete sequences	92 %

*Quality of assembled transcriptome was evaluated using Transrate v2.14.0 and BUSCO v5.2.2 against the lineage of arthropoda_odb10. Read mapping rate was estimated by mapping reads from each library to the CTen_1 reference genome (GenBank assembly GCA_030545055.1) using Hisat2 v2.2.1.

### Read mapping and differential gene expression analysis

The expression count matrix was generated by mapping reads from each sample against the assembled transcriptome using Salmon v1.10.1 [30]. Differentially expressed genes between viruliferous and non-viruliferous BLHs were identified using DESeq2 v3.16 [31]. Only transcripts with an absolute log_2_ fold change ≥1 and false discovery rate (FDR)-corrected *p*-value < 0.05 were considered differentially expressed. The raw count data were normalized by variance stabilizing transformation (VST) function in DESeq2. The biological variations between samples were assessed by principal component analysis in R (4.2.1) using the VST-normalized count data (genes with zero expression values across all samples were excluded). Heatmaps in this study were generated based on VST-normalized count values by employing average linkage clustering method and Pearson’s correlation for distance measurement using pheatmap v1.0.12 R package (RRID : SCR_016418).

### Gene ontology classification and enrichment analysis

Differentially expressed sequences were annotated against the NCBI non-redundant protein databases with Blastx and an E-value cut-off of 10^−5^ using OmicsBox v2.2 [32]. Sequences assigned with Gene Ontology (GO) terms were classified into three GO categories: biological process (BP), molecular function (MF) and cellular component (CC). GO enrichment analysis was performed for up- and downregulated transcripts using a two-tailed Fisher’s exact test with an FDR cut-off of 0.05 in OmicsBox. The entire BLH transcriptome was annotated as described above and used as background gene set for the GO enrichment analysis.

### Validation of RNA-Seq using reverse transcription-quantitative PCR (RT-qPCR)

The aliquots of RNA samples used for RNA-Seq were tested by RT-qPCR to validate the observed changes in gene expression for differentially expressed transcripts (DETs). Primer sets targeting the eight selected DETs and two BLH reference genes, actin and RPL13, were listed in Table S1. The two reference genes were identified from the BLH transcriptome assembled in this study. cDNA synthesis utilized 500 ng of RNA using the Verso cDNA Synthesis Kit (Thermo Scientific, Waltham, MA), following the manufacturer’s instructions, and was subsequently diluted fourfold in nuclease-free water. Quantitative PCR analysis was performed in duplicate using SYBR green super mix (Bio-Rad, Hercules, CA) and CFX Connect Real Time instrument (Bio-Rad). Each reaction mix (10 µl) was prepared per manufacturer instructions with 2 µl cDNA template for each primer set. Thermocycling conditions consisted of 95 °C for 30 s, followed by 40 cycles of 95 °C for 10 s, 55 °C for 30 s, 72 °C for 30 s, with a final melt curve of 95 °C for 30 s and 65 °C for 5 s increasing to a final temperature of 95 °C. Primer specificity was determined by gel electrophoresis and melt curve (Fig. S3). The expression level of each transcript was normalized to both actin and RPL13 reference genes [33] and the log_2_-transformed fold change was calculated by comparing viruliferous BLH against non-viruliferous control. Pearson’s correlation analysis was performed on the log_2_-fold changes derived from RT-qPCR and RNA-Seq using GraphPad Prism software. A one-tailed hypothesis test was used to assess the null hypothesis of no positive relationship between RT-qPCR and RNA-Seq (*p*-value < 0.05).

## Results

### Effect of BCTV on BLH performance

**Fig. 1.**
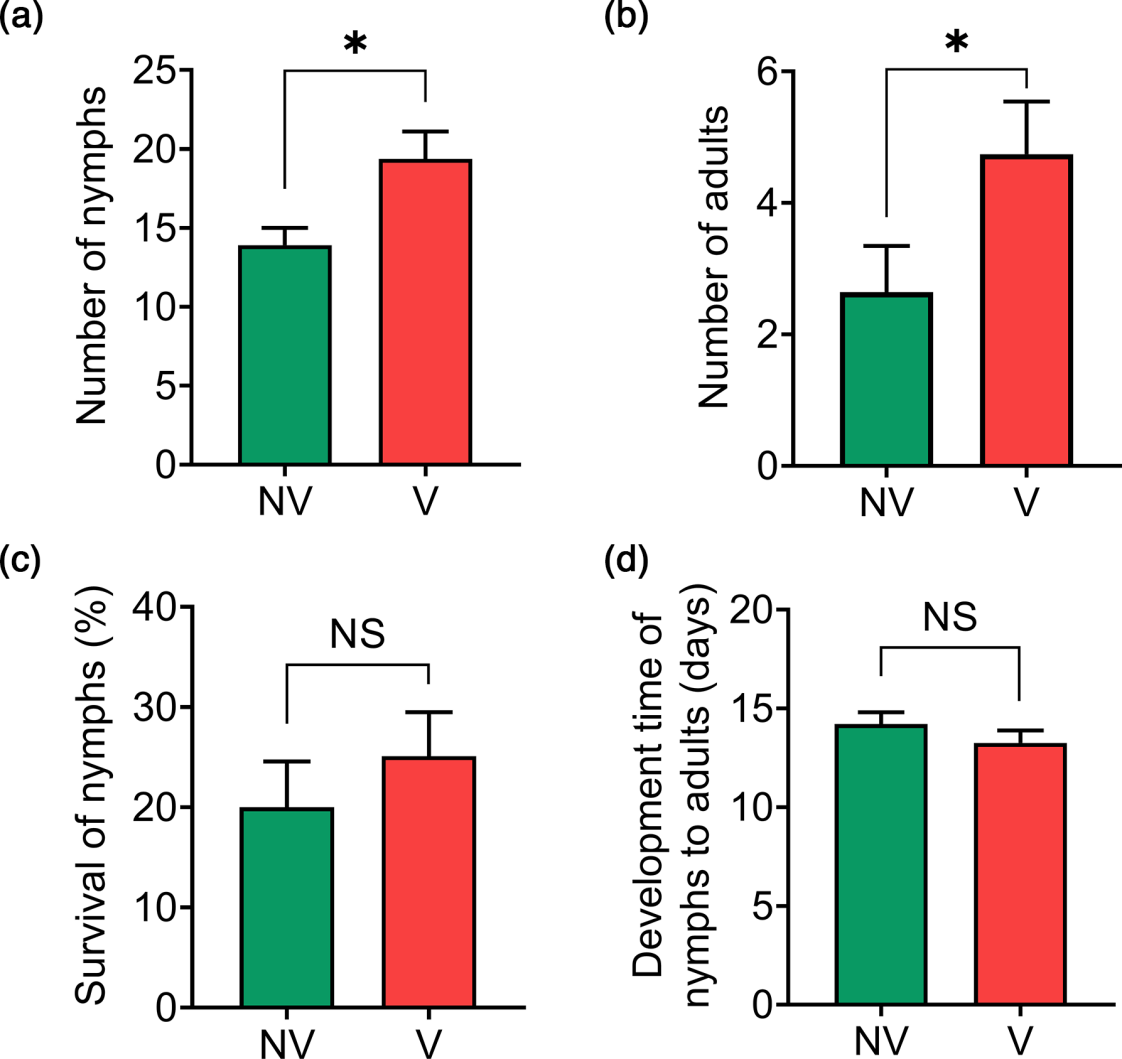
Performance of beet curly top virus-viruliferous and non-viruliferous BLHs on sugar beet plants. Six viruliferous (V) or non-viruliferous (NV) BLHs (four females and two males) were allowed to feed and oviposit on a sugar beet plant. The number of offspring nymphs, (**a**) number of emerging adults from nymphs, (**b**) survival of nymphs (**c**) and development time of nymphs to adults (**d**) were examined. A total of 39 and 27 biological replicates (individual plant/cages) were included for viruliferous and non-viruliferous insects, respectively. Asterisk (*) and NS indicate significant (*P<*0.05) and insignificant differences between treatments, respectively.

Virus infection had a significant effect on BLH performance. Viruliferous adult BLH produced a significantly higher number of nymphs on average (19.4±9.02) compared to non-viruliferous BLH (13.9±6.90) (Fig. 1a). The increased number of nymphs from viruliferous BLHs resulted in a 44 % higher number of surviving adults (4.7±4.16) than non-viruliferous BLHs (2.6±4.40) (Fig. 1b). The number and developmental time of surviving offspring were also estimated to examine potential maternal effects linked to BCTV infection. There was no difference in survival rate (viruliferous, 0.25±0.23 vs non-viruliferous, 0.20±0.28) and developmental time of nymphs (viruliferous, 9.81±6.39 vs non-viruliferous, 8.74±7.37) produced by viruliferous and non-viruliferous BLH (Fig. 1c, d).

### BLH transcriptome assembly

A total of 26.56 billion paired-end reads were obtained from all BLH samples after quality and adaptor trimming. Each sample contained more than 2.53 billion reads with Q30 quality scores≥90 % (Table S2). The high-quality reads from each library were used for genome-guided transcriptome assembly. A total of 63 534 transcripts were assembled with an average length of 1 979 bp (Table 1). Across the libraries, 87–89 % of reads were successfully mapped to the CTen_1 reference genome, among which 69–72 % were aligned uniquely. The BUSCO analysis confirmed that 92 % of evolutionarily conserved gene content in the Arthropoda odb10 database (www.orthodb.org) was identified in our transcriptome (Table 1).

### Transcriptomic response of BLH to BCTV infection

**Fig. 2.**
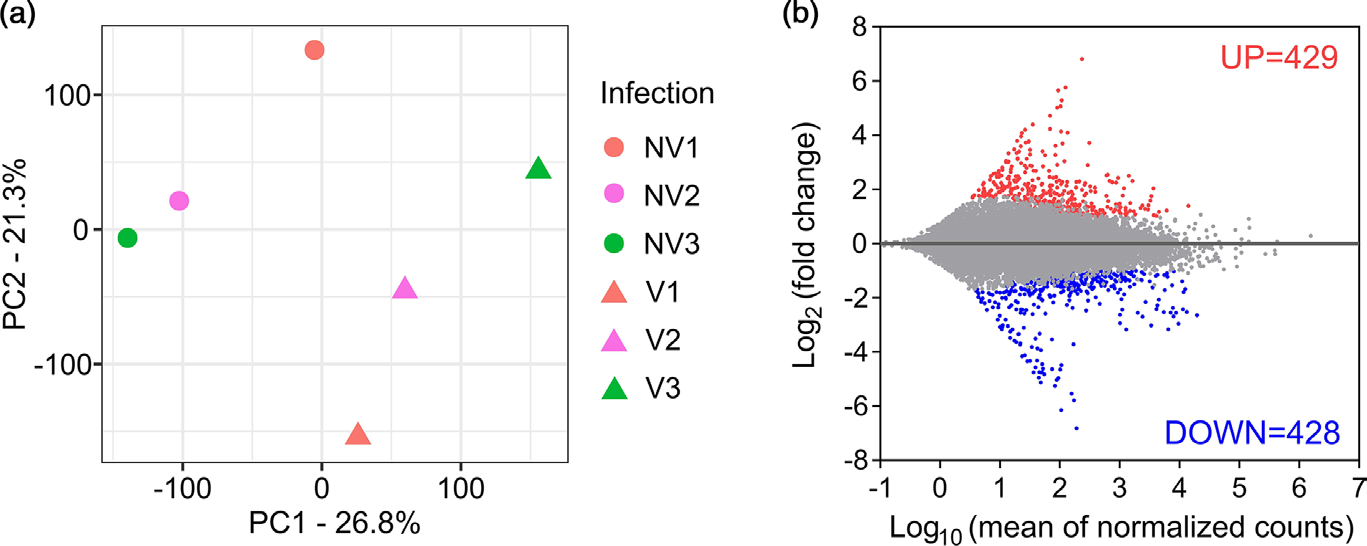
Global transcriptional responses of BLHs to beet curly top virus infection. (**a**) Principal component analysis of normalized count data from all samples. Three biological replicates were included for viruliferous (V) and non-viruliferous (NV) BLHs. (**b**) DETs induced by beet curly top virus infection. Differential gene expression analysis was performed using DESeq2 package. Transcripts were considered differentially expressed when the absolute log_2_-fold changes (V against NV) were ≥1 with false discovery rate <0.05.

The biological variations between viruliferous and non-viruliferous BLH samples were evaluated by the principal component analysis (PCA). The first two principal components (PC1 and PC2) accounted for 48.1 % of total variation across the samples. Overall, the viruliferous and non-viruliferous BLH samples formed two groups and were separated from each other by PC1 and PC2 (Fig. 2a), suggesting that virus infection is attributed to the observed variation between these two sample clusters. It is worth noting that we did not detect any BCTV reads in either the viruliferous or non-viruliferous samples, which indicates that the virus may not actively transcribe its genes in the vector.

The differential gene expression analysis identified a total of 857 DETs in BLHs in response to BCTV infection (Fig. 2b). A comparable number of up- (429) and downregulated transcripts (428) were observed with an averaged log_2_-fold change of 1.883 and −1.880, respectively. Heatmap clustering of all DETs confirmed the distinct patterns of gene regulation between viruliferous and non-viruliferous BLHs (Fig. S2). Within the top 10 most upregulated transcripts, many showed their provisional functions in fatty acid biosynthesis, oogenesis, progression of the mitotic cell cycle, modification of chromatin and signal transduction (Table 2). In contrast, the top 10 most downregulated transcripts were primarily associated with muscle contraction and stability, proteolysis, regulation of transcription and translation and DNA replication and repair (Table 2).

**Table 2.** Top 20 most DETs in BLHs in response to beet curly top virus infection

Transcript ID	Log_2_ fold change	Adj. *p*-value	Gene description	Predicted biological function
MSTRG.5247.10	8.339	7.46E-90	Acyl-CoA Delta(11) desaturase-like	Unsaturated fatty acid biosynthetic process
MSTRG.6904.2	6.808	9.18E-48	Heterochromatin protein 1-like isoform X1	Negative regulation of transcription; chromosome organization; negative regulation of cellular macromolecule biosynthetic process
ANN02543-RA	5.758	4.94E-29	GNAT domain, acyl-CoA N-acyltransferase, FR47-like	na
MSTRG.9037.1	5.653	1.28E-28	Serine/arginine repetitive matrix protein 2-like isoform X4	na
ANN19788-RA	5.289	1.33E-22	Protein gustavus isoform X2	Intracellular signal transduction; proteasome-mediated ubiquitin-dependent protein catabolic process
ANN17804-RA	5.066	5.48E-20	Tetraspanin-18-like	na
MSTRG.16665.3	5.016	1.07E-19	Hypothetical protein J6590_041573	na
MSTRG.6946.1	4.715	6.06E-17	Pogo transposable element with ZNF domain	Regulation of transcription; regulation of cellular macromolecule biosynthetic process
ANN34708-RA	4.396	8.09E-15	GNAT domain, acyl-CoA N-acyltransferase, FR47-like	na
MSTRG.12393.23	4.226	1.20E-12	na	na
ANN22164-RA	−4.952	1.33E-20	FACT complex subunit spt16 isoform X1	DNA replication; DNA repair; positive regulation of transcription elongation; chromosome organization; regulation of cellular macromolecule biosynthetic process
ANN15214-RA	−4.965	6.95E-19	3-Ketoacyl-CoA thiolase, mitochondrial	Biosynthetic process of amino acids, sugars and glycoproteins
ANN31278-RA	−5.034	9.04E-20	Malate dehydrogenase, cytoplasmic	Tricarboxylic acid cycle; malate metabolic process
ANN05891-RA	−5.126	7.60E-23	WD repeat-containing protein 18	DNA biosynthetic process; rRNA processing; cellular macromolecule biosynthetic process
ANN21089-RA	−5.259	9.75E-23	NADH dehydrogenase [ubiquinone] 1 beta subcomplex subunit 10	na
MSTRG.15671.5	−5.549	7.52E-25	Low-quality protein: uncharacterized protein LOC124361938	na
ANN23947-RA	−5.785	5.19E-28	Pumilio homolog 3	Translational elongation; regulation of translation
ANN01521-RA	−6.151	5.82E-38	Carboxypeptidase E	Peptide metabolic process; protein processing
MSTRG.15779.5	−6.823	6.87E-50	Titin isoform X3	Homophilic cell adhesion via plasma membrane adhesion molecules; cell recognition
MSTRG.1821.4	−9.661	6.44E-135	Troponin T isoform X2	Regulation of muscle contraction; sarcomere organization

### Gene Ontology enrichment analysis of DETs

**Fig. 3.**
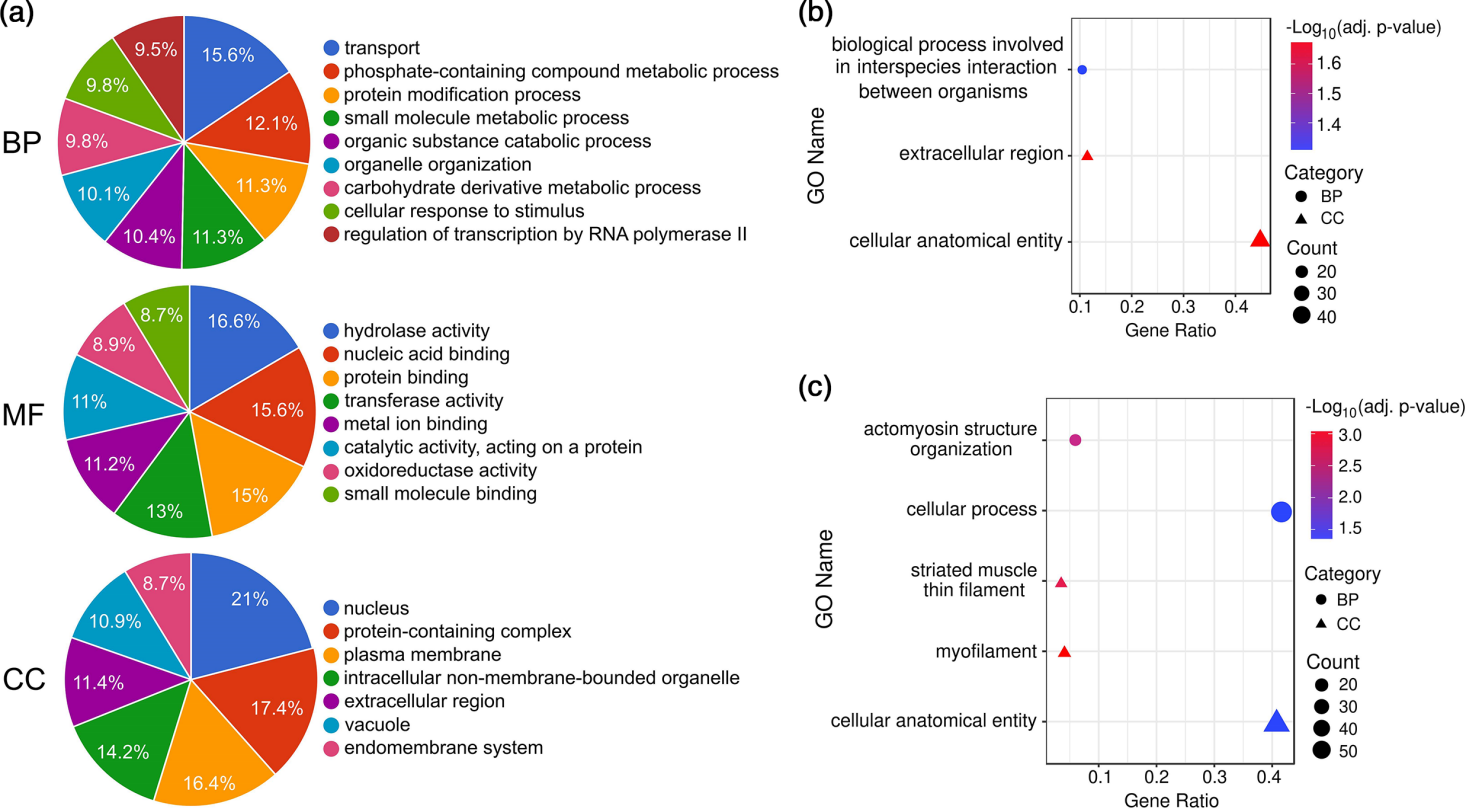
GO analysis of DETs. (**a**) All annotated DETs were classified into BP, MF and CC. (**b**) Enriched GO terms in the upregulated transcripts. (**c**) Enriched GO terms in the downregulated transcripts. The size and colour of the dots represent the number of sequences associated with the GO term and the Benjamini-Hochberg adjusted *p*-values, respectively.

Of 857 DETs, 61 % were annotated with GO terms and classified into three functional categories, including the BP (394), MF (362) and CC (509). Among these annotated DETs, the most prominent GO annotations in BP category were ‘transport’, ‘phosphate-containing compound metabolic process’, ‘protein modification process’ and ‘small molecule metabolic process’, which accounted for 50.3 % of sequences in this category (Fig. 3a). As for DETs assigned to MF category, more than half (60.2 %) were associated with ‘hydrolase activity’, ‘nucleic acid binding’, ‘protein binding’, and ‘transferase activity’ (Fig. 3a). In the CC category, 54.8 % of sequences were involved in the ‘nucleus’, ‘protein-containing complex’ and ‘plasma membrane’ (Fig. 3a). Among all identified DETs, 13.1 % were shown as hypothetical/uncharacterized proteins, and 26.7 % were predicted to be BLH-specific as they had no sequence matches in the currently available NCBI nr protein databases (Table S3).

GO enrichment analysis revealed that the upregulated DETs were enriched in GO terms associated with ‘biological process involved in interspecies interaction between organisms’, ‘cellular anatomical entity’ and ‘extracellular region’ (Fig. 3b). On the contrary, the enriched GO terms for downregulated DETs were associated with ‘actomyosin structure organization’, ‘cellular process’, ‘myofilament’, ‘cellular anatomical entity’ and ‘striated muscle thin filament’ (Fig. 3c).

**Fig. 4.**
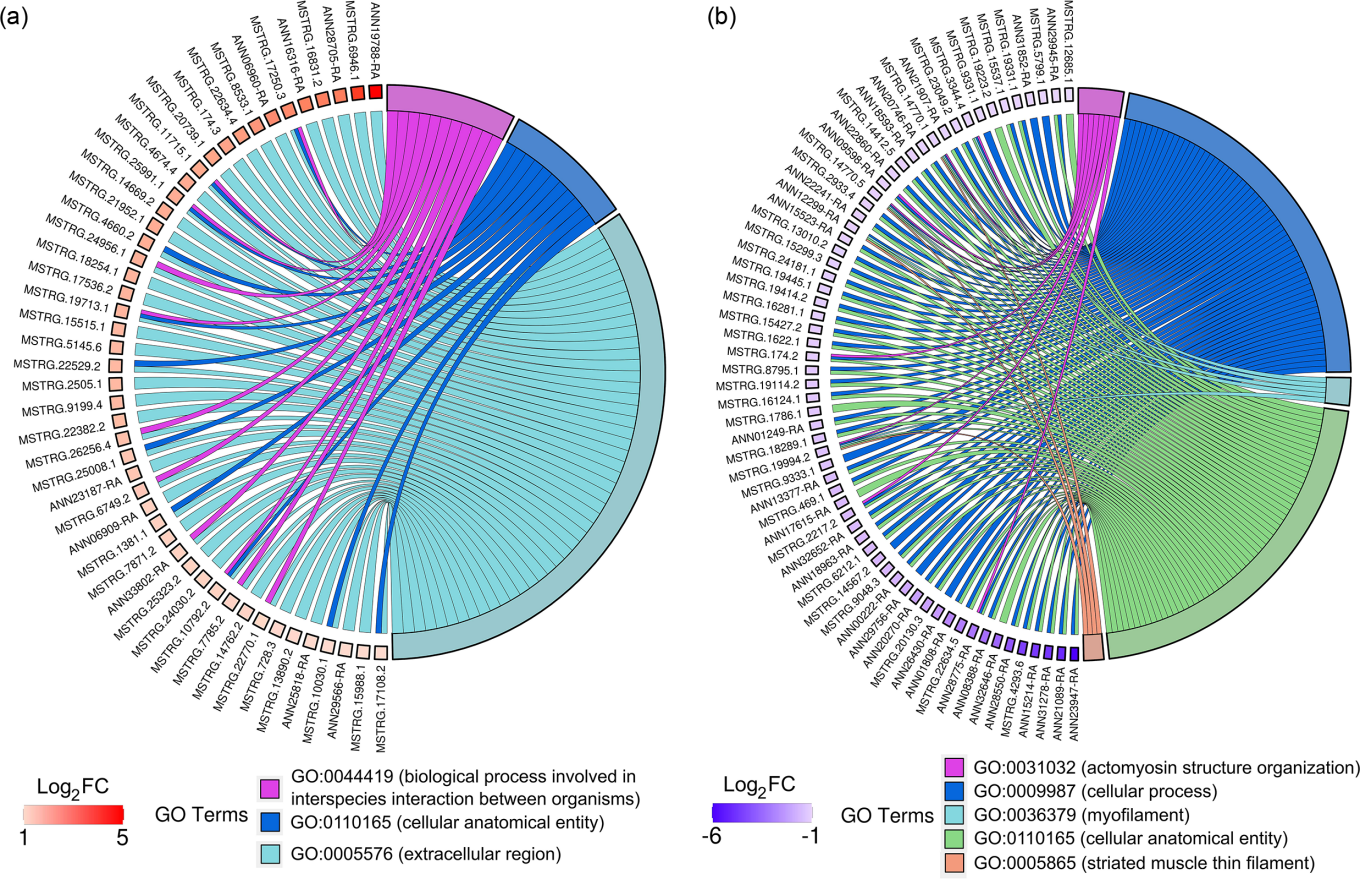
DETs associated with enriched GO terms. Sankey plots present the significantly enriched GO terms associated with up- (**a**) and downregulated (**b**) transcripts. The colour of gene nodes indicates the magnitude of log_2_-fold change (viruliferous vs non-viruliferous).

Transcripts upregulated within the enriched GO-BP category were mainly represented by proteins involved in embryogenesis, spermatogenesis, reproduction and cell morphogenesis/migration/death, including akirin-2, inactive peptidyl-prolyl cis-trans isomerase FKBP6, serpin B12 and serine/threonine-protein kinase tousled-like 2 isoform X3 (Fig. 4a). Additionally, proteins involved in insect innate immunity were also enriched in the same GO term, such as peptidoglycan-recognition protein LB-like, serine protease inhibitor 88Ea-like, beta-1,3-glucan-binding protein and peptidoglycan-recognition protein SC2-like (Fig. 4a).

In contrast, the predominant transcripts downregulated within the enriched GO-BP category were encoding proteins involved in cytoskeletal structures and functions (e.g. troponin I isoform X8 and X2, myophilin and titin isoform X9) (Fig. 4b), suggesting the potential reduction in muscle contraction, cell motility, cytokinesis and/or organelle transport.

### Differentially expressed transcripts associated with reproduction

**Fig. 5.**
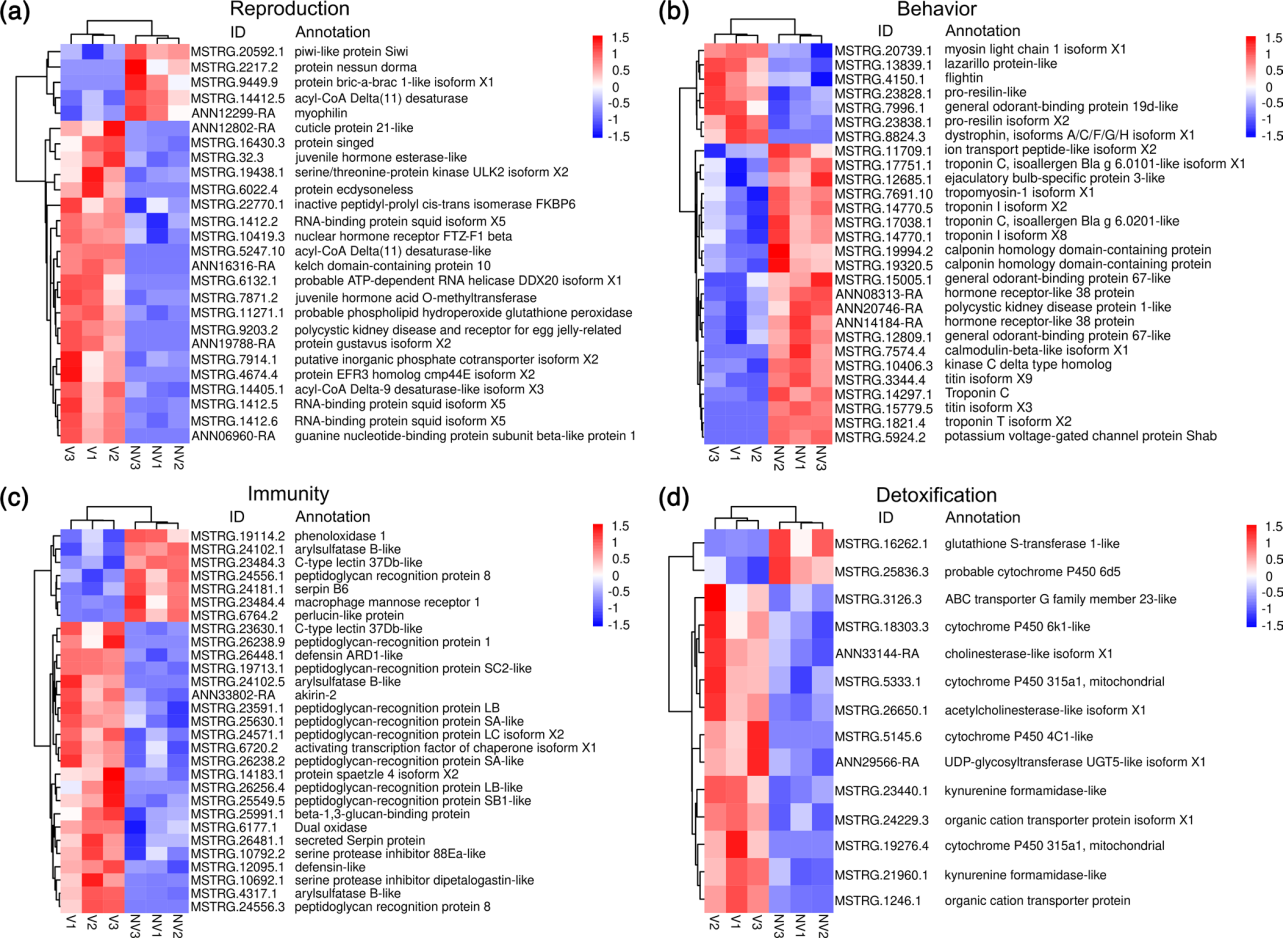
Heatmaps showing the expressions of DETs related to (a) reproduction, (**b**) behaviour, (**c**) immunity and (d) detoxification. Expression values are Z-score transformed across all samples. Colour key indicates higher (red) to lower (blue) expression levels. Rows and columns indicate transcripts and insect samples from three biological replicates. NV and V indicate non-viruliferous and viruliferous BLHs.

Further classification of the annotated DETs revealed that 26 showed their provisional functions in development, viability and fertility of germline and embryos (Fig. 5a). Among them, 81 % were upregulated in viruliferous BLHs compared to non-viruliferous controls. Seven DETs were identified to be associated with oogenesis, and all except one (protein bric-a-brac 1-like isoform X1) were upregulated in viruliferous BLHs, including protein gustavus isoform X2, nuclear hormone receptor FTZ-F1 beta, putative inorganic phosphate cotransporter isoform X2, juvenile hormone acid O-methyltransferase, serine/threonine-protein kinase ULK2 isoform X2, protein singed and protein ecdysoneless. All three DETs linked to embryogenesis were identified to be upregulated in viruliferous BLHs, including protein EFR3 homolog cmp44E isoform X2, probable ATP-dependent RNA helicase DDX20 isoform X1 and guanine nucleotide-binding protein subunit beta-like protein 1. Additionally, four DETs involved in sperm development, morphogenesis, integrity and fertility were upregulated in viruliferous BLHs, including polycystic kidney disease and receptor for egg jelly-related protein-like isoform X2, probable phospholipid hydroperoxide glutathione peroxidase isoform X1, kelch domain-containing protein 10 and inactive peptidyl-prolyl cis-trans isomerase FKBP6. We also found that DETs related to vitellogenesis and germ cell maintenance were upregulated in viruliferous BLHs.

### Differentially expressed transcripts associated with behaviour

There were 28 DETs found to be involved in insect behaviour, including muscle movement, locomotor activities, mating and sensory perception (Fig. 5b). Within the 17 DETs linked to muscle movement and stability, including those in the troponin protein family, titin proteins and calponin protein, 82 % were downregulated in viruliferous BLHs. This aligns with the finding of our GO enrichment analysis, which revealed significantly enriched GO terms associated with cytoskeletal structures and functions. Moreover, four flight-related DETs were identified, including two upregulated pro-resilin proteins, one downregulated kinase C delta type homolog and one downregulated ion transport peptide-like isoform X2. Additionally, six DETs relating to odour perception and sexual behaviour were identified, comprising three general odorant-binding proteins (one upregulated and two downregulated), one downregulated ejaculatory bulb-specific protein 3-like and two downregulated hormone receptor-like 38 proteins.

### Differentially expressed transcripts associated with innate immunity

There were 29 identified DETs associated with insect innate immunity (Fig. 5c). Among them, 22 were upregulated in viruliferous BLHs when compared to non-viruliferous insects. There were 15 DETs showed to be involved in recognition of invading microorganisms, with 80 % exhibited significant upregulation in viruliferous BLHs, such as beta-1,3-glucan-binding protein, C-type lectin 37Db-like, macrophage mannose receptor 1 and peptidoglycan recognition proteins. Moreover, 12 DETs were determined to be associated with Toll and IMD pathways, melanisation processes and antimicrobial activities, including protein spaetzle 4 isoform X2, akirin-2, secreted serpin protein, arylsulfatase B-like and defensin ARD1-like. Almost all of these DETs, except two, showed significant upregulation in viruliferous BLHs.

### Differentially expressed transcripts associated with detoxification

A total of 14 DETs were shown to have functions in detoxification of xenobiotic and endogenous harmful metabolites in insects (Fig. 5d). Among them, 86 % were upregulated in viruliferous BLHs, covering several major detoxification gene families. Of five DETs encoding cytochrome P450 proteins, four showed 2.2- to 4.2-fold upregulation in viruliferous BLHs. One ABC transporter G family member 23-like, two organic cation transporter proteins and one UDP-glycosyltransferase UGT5-like isoform X1 were also identified to be significantly upregulated in viruliferous BLHs. Other upregulated DETs involved in metabolism of endogenous toxic compounds include kynurenine formamidase-like, cholinesterase-like isoform X1 and acetylcholinesterase-like isoform X1. The glutathione S-transferase 1-like and probable cytochrome P450 6d5 were the only two downregulated DETs in viruliferous BLHs.

### Differentially regulated transcripts associated with energy, lipid, protein synthesis and amino acid metabolisms

**Fig. 6.**
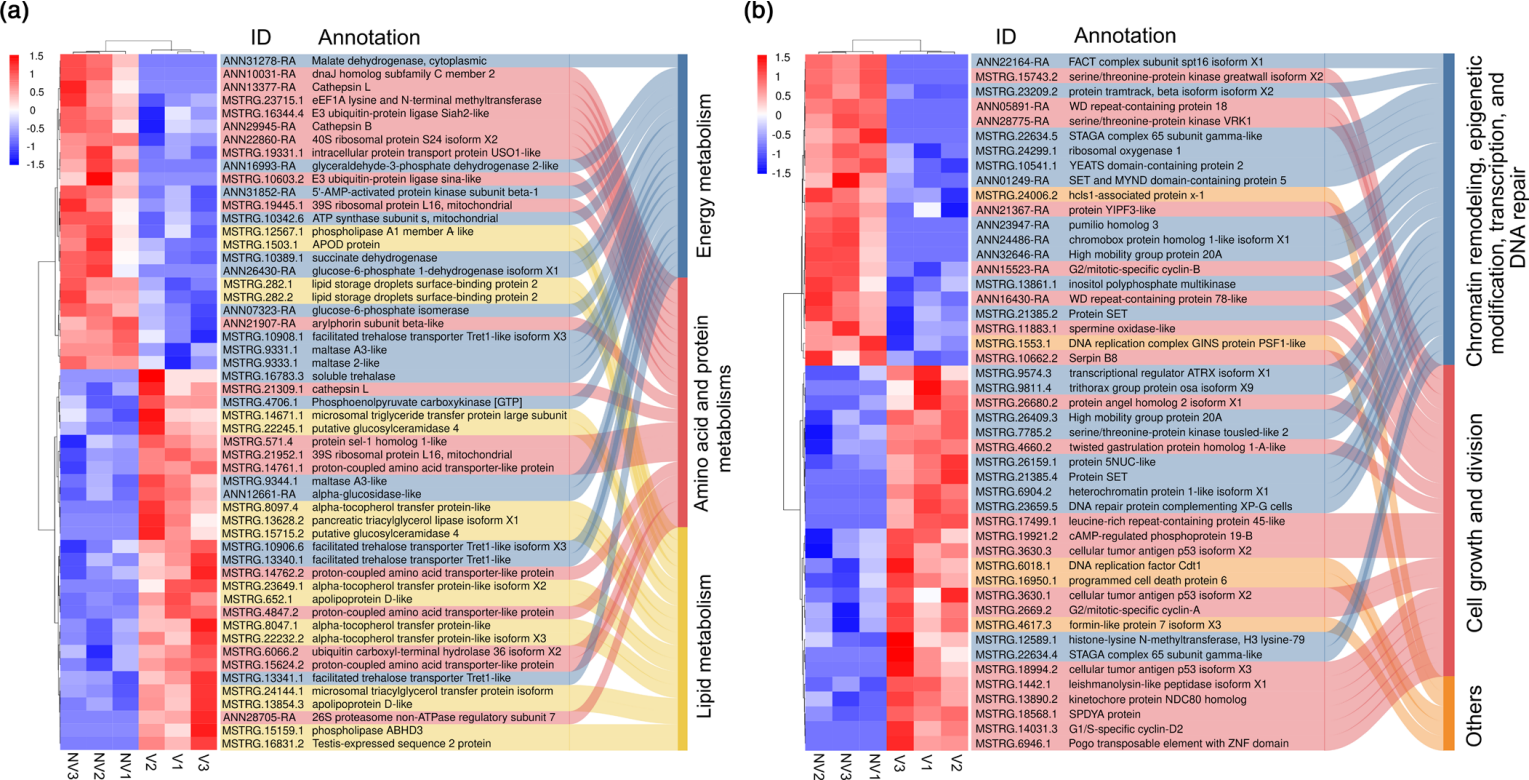
Heatmaps showing the expressions of DETs related to (a) energy, lipid, amino acid and protein metabolisms and (b) cellular function. Expression values are Z-score transformed across all samples. Colour key indicates higher (red) to lower (blue) expression levels. Rows and columns indicate transcripts and insect samples from three biological replicates. NV and V indicate non-viruliferous and viruliferous BLHs.

Compared to non-viruliferous BLHs, there were a total of 53 DETs associated with energy, lipid, amino acid and protein metabolisms (Table S3). Among these DETs, 55 % exhibited upregulation in viruliferous BLHs. Specifically, 41 % of transcripts associated with energy metabolism demonstrated increased expression in viruliferous BLHs, with a focus on key players in glucose metabolism and trehalose transport, including soluble trehalase, alpha-glucosidase and the facilitated trehalose transporter Tret1 (Fig. 6a). Examining downregulated DETs in energy metabolism, several were linked to carbohydrate dissimilation, proteolysis and energy balance and production. These included maltase A3-like, glyceraldehyde-3-phosphate dehydrogenase 2-like, 5′-AMP-activated protein kinase subunit beta-1 and two tricarboxylic acid cycle-related transcripts, namely malate dehydrogenase and succinate dehydrogenase. For lipid metabolism, 76 % of DETs were upregulated in viruliferous BLHs, primarily functioning in lipid digestion and transport (Fig. 6a). Notable examples include phospholipase ABHD3, pancreatic triacylglycerol lipase isoform X1, apolipoprotein d-like and microsomal triglyceride transfer protein. Regarding amino acid and protein metabolisms, 19 DETs were identified, with 47 % exhibiting upregulation (Fig. 6a). These DETs primarily participated in protein degradation and amino acid transport, including 26S proteasome non-ATPase regulatory subunit, cathepsin L and proton-coupled amino acid transporter protein. In contrast, most downregulated DETs in this category were involved in protein synthesis, folding and storage, such as dnaJ homolog and arylphorin subunit beta-like.

### Differentially regulated transcripts associated with cellular function

There were 47 DETs identified to play a substantial role in cellular function, most notably with cell growth and division, epigenetic modification of chromatin and DNA repair (Table S2). Overall, there was a comparable number of up- (26) and downregulated DETs (21) (Fig. 6b). Of 47 DETs, a great majority of them (42) were associated with chromatin remodelling, transcription, cell cycle control and proliferation and DNA repair, with 55 % upregulated in viruliferous BLHs. DETs were also associated with apoptosis, DNA replication and cell morphology, including programmed cell death protein 6, hcls1-associated protein x-1, DNA replication factor Cdt1 and formin-like protein 7 isoform X3.

### Validation of RNA-Seq analysis by RT-qPCR analysis

**Fig. 7.**
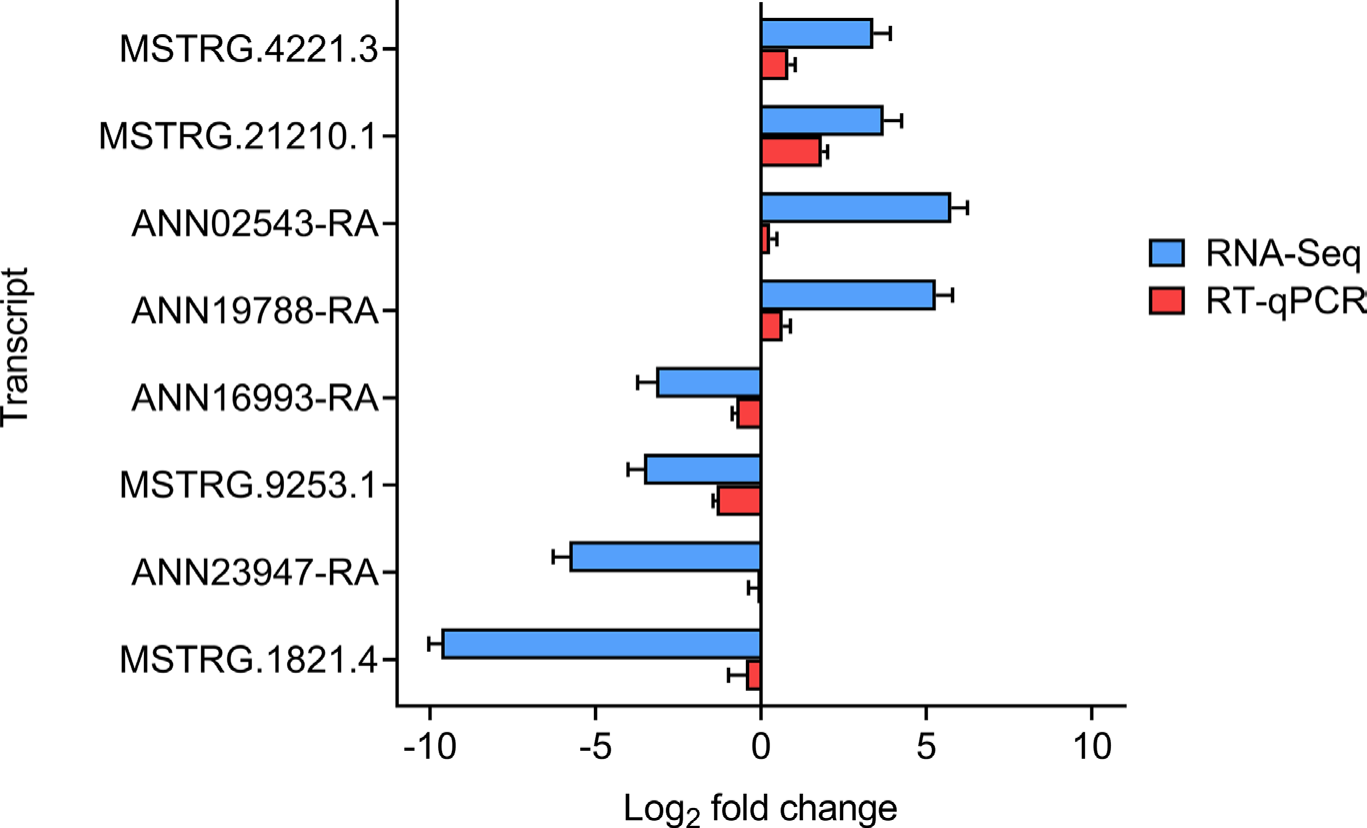
Validation of differential gene expression analysis using RT-qPCR. Expression levels of eight DETs of BLHs in response to beet curly top virus infection were detected using RT-qPCR. The expression of each transcript was normalized using two BLH reference genes, actin and RPL13, and then calibrated relative to non-viruliferous BLHs. Fold change of each transcript (viruliferous against non-viruliferous) was log_2_-transformed. Each bar represents the mean±standard error of three biological replicates.

Eight selected DETs were validated using RT-qPCR to confirm the fold changes observed in the RNA-Seq data (Fig. 7, Table S4 and S5). The magnitude of fold changes detected by the RNA-Seq was overall higher than those by RT-qPCR, which could be due to the methodological difference in detection sensitivity between RT-qPCR and RNA-Seq. The significant Pearson’s correlation (coefficient=0.6739, *p*-value=0.0334) of the fold changes further verify the RNA-Seq analysis.

## Discussion

There is extensive evidence that plant viruses manipulate vector performance and behaviour to optimize virus transmission (reviewed in [13]). Additionally, accumulating evidence points to transcriptional responses that underline virus-induced changes in the vector (reviewed in [34]). In this study, we delved into the relationship between BCTV and its insect vector, BLH, aiming to unravel the underlying mechanisms influencing vector performance. Our investigation revealed a significant impact of BCTV on BLH performance, with viruliferous insects showing increased nymphal production compared to non-viruliferous counterparts. This phenomenon led to a substantial elevation in the number of surviving adults among viruliferous BLHs, indicative of a potential mechanism enhancing virus transmission. To elucidate the molecular basis of these observations, we conducted a comprehensive transcriptomic analysis of BLHs in response to BCTV acquisition. To our knowledge, this study is the first to unveil the global transcriptional pathways modulated in the BLH following BCTV acquisition, providing new insights into key molecular players associated with virus-vector interactions.

### Reproduction, development and fitness-related transcripts

A great majority of DETs (81 %) related to development, viability and fertility of germline and embryos were upregulated in viruliferous BLHs compared to non-viruliferous controls. Specifically, transcripts related to oogenesis and embryogenesis were upregulated in viruliferous BLHs. This finding is intriguing because BCTV is not transovarially transmitted. Other studies have found that viruses could promote oogenesis in planthoppers to increase the frequency of transovarial transmission [35,36]. Additionally, DETs involved in sperm development, morphogenesis, integrity and fertility were upregulated in viruliferous BLHs. Moreover, DETs related to vitellogenesis, such as juvenile hormone acid O-methyltransferase, showed increased expression in viruliferous BLHs. Juvenile hormone is known to stimulate yolk production (vitellogenesis) in female ovaries and promote accessory gland growth and the production of accessory gland secretion in males [37]. Upregulation of vitellogenin-related genes has been observed in the whitefly *Bemisia tabaci* Mediterranean (MED) biotype that fed on tomato yellow leaf curl virus (TYLCV)-infected, tomato chlorosis virus (ToCV)-infected or TYLCV and ToCV co-infected tomato plants [38].

Several studies have demonstrated that geminiviruses, namely begomoviruses, can elicit positive, neutral or negative effects on their whitefly vectors, depending on specific virus-whitefly combinations. For example, the tomato yellow leaf curl China virus (TYLCCNV) had varying impacts on the indigenous (ASIA II 3) and invasive (MEAM1) whitefly biotypes, with significant increases in the number of oocytes and eggs laid by MEAM1 whiteflies feeding on TYLCCNV-infected tobacco plants, while no significant differences were observed between ASIA II 3 whiteflies feeding on TYLCCNV-infected and non-infected plants [39]. Additionally, the MED whitefly biotype performed better on virus-infected tomato than on uninfected controls [40]. Another study found that feeding on BCTV-infected sugar beet plants prolonged the development time of both male and female nymphs of BLHs by an additional 1–10 days compared to those on uninfected plants [24]. Our study further revealed that viruliferous BLHs produced a significantly higher number of nymphs, resulting in a 44 % increase in maturing adults compared to non-viruliferous BLHs, potentially contributing to virus transmission. The observed higher fecundity in viruliferous insects was supported by a concomitant upregulation of transcripts related to development, viability and fertility of germline and embryos in viruliferous insects. Collectively, these findings provide convincing evidence of upregulation of reproductive and development-related genes in virus-mediated modulation of vector fitness in virus-vector interactions.

While our study investigated the transcriptional changes in BLHs following BCTV acquisition, the impact of the virus on its vector may result from a direct effect of the virus on the vector or an indirect plant-mediated effect. Plant-mediated effects may occur through modifications in plant primary and secondary metabolism, such as phytohormones, which can alter vector performance and behaviour in ways that favour virus propagation (reviewed in [41]). Further investigation is warranted to disentangle the direct effects of the virus from the indirect effects on BLH responses.

### Behaviour, movement and sensory perception

In contrast to transcripts related to reproduction, most DETs (82 %) associated with insect behaviour, including muscle movement, locomotor activities, mating and sensory perception, were downregulated in viruliferous BLHs. For example, troponin C, tropomyosin and troponin I all play a central role in the calcium-dependent regulation of muscle contractions [42] and were downregulated in viruliferous BLHs. In addition to these, myosin light chain I and kinase C delta type homolog, which are involved in flight, were also downregulated. This suggests a possible trade-off between reproduction and mobility in viruliferous insects, leading to an increased vector population and reduced dispersal. Numerous studies have shown the life-history trade-off between fecundity and dispersal capability in various insect species, including *Spodoptera frugiperda*, *Aphis glycines*, *Oscinella frit* and *Stenobothrus lineatus*, and several wing-dimorphic species, where increased dispersal is associated with reduced investment in reproduction [43,49]. However, additional research is necessary to explore the existence of the trade-off and the extent to which reduced mobility in viruliferous BLHs impacts virus transmission.

To date, there is limited evidence regarding the impact of BCTV on the behaviour of the BLH [7]. Recently, Lee and colleagues showed that non-viruliferous BLHs preferentially probed on barley (BCTV non-host) and ribwort plantain (BCTV host) compared with tomato (BCTV host), whereas viruliferous BLHs showed no preference. This change in probing preference in viruliferous BLHs may result in enhanced BCTV transmission in tomato fields, which was supported by simulation modelling [25]. Additionally, Stafford *et al.* characterized different probing waveforms associated with the feeding behaviour of BCTV-viruliferous BLH using electrical penetration graph. However, the impact of BCTV on BLH’s feeding behaviour remains unclear as they did not compare the feeding activities between viruliferous and non-viruliferous insects [50]. While our study did not examine the movement or probing preference and activity of BLH after virus infection, it is possible that these transcripts could alter the behaviour of viruliferous BLHs, as many of these genes have been linked to locomotion in other insects [51,52]. Furthermore, related to sensory perception activity, one transcript encoding general odorant-binding protein 67-like was downregulated, whereas another general odorant-binding protein 19-like transcript was upregulated. These odorant-binding proteins can play pivotal roles in the olfactory system and are essential for mating and oviposition host selection in more than 100 insect species [53]. Changes in their expression may similarly alter the behaviour of BLHs.

### Immunity, stress and detoxifying response

The innate immune system is the major defence mechanism used by insects to fight against foreign invaders, such as viruses [54,55]. A majority of the immune response genes were upregulated in viruliferous BLHs when compared to non-viruliferous insects. For example, DETs involved in recognition of invading microorganisms, such as beta-1,3-glucan-binding protein, C-type lectin 37Db-like, macrophage mannose receptor 1 and peptidoglycan recognition proteins, were upregulated. Moreover, DETs associated with Toll and IMD pathways, melanization processes and antimicrobial activities showed upregulation in viruliferous BLHs. This might lead to a decrease of viral particles within the body of the viruliferous BLHs [56,60]. Other transcriptomic studies demonstrated that immune responses were activated in the MEAM1 whitefly when fed on TYLCV-infected, ToCV-infected or TYLCV and ToCV co-infected plants [38,61]. In addition, stress response-related transcripts were upregulated in viruliferous BLHs. These included several cytochrome P450s, ABC transporters and acetylcholinesterase that are important in the metabolism of xenobiotics, such as plant allelochemicals and insecticides. In contrast to this study, most transcripts encoding detoxification enzymes were downregulated in virus-infected whiteflies [38,61]. These results may suggest that the susceptibility of BLHs to insecticides may be altered by viral infection.

### Virus-induced metabolism and other cellular functions

The majority of DETs associated with energy or carbohydrate metabolism were upregulated, including those encoding glucosidases and proteins involved in trehalose transport. Glucosidases are mainly involved in the hydrolysis of carbohydrates; they also play important roles in normal cellular function and pathogen defence [62]. Expression of glucosidase genes in the MED whitefly was also altered by TYLCV infection, ToCV infection or TYLCV and ToCV co-infection [38]. Lipids play critical roles in energy homeostasis, membrane structure and signalling [63] and are considered a key feature of cellular changes associated with viral infection [38,61, 64, 65]. Most genes in this study associated with lipid metabolism were upregulated. This contrasts with the MED whitefly, where most of the lipid metabolism genes were downregulated in response to TYLCCNV infection [61] and TYLCV infection, ToCV infection and TYLCV and ToCV co-infection [38]. Similarly, genes involved in lipid, carbohydrate and amino acid metabolism were downregulated in other whitefly species, MEAM1, after feeding on TYLCV-infected tomato plants [66]. Such metabolic suppression may have led to reductions in fecundity and longevity in the MEAM1 whitefly [67]. In our study, there were similar numbers of DETs associated with amino acid and protein metabolism. DETs participating in protein degradation, such as 26S proteosome and ubiquitin carboxyl-terminal hydrolase 36 isoform X2, were upregulated. The alteration of these pathways was also reported in other virus-vector systems and led to enhanced viral nucleoprotein accumulation and titre [68]. In contrast, DETs, such as cathepsins, 40S ribosomal protein and E3 ubiquitin-protein ligase, were downregulated. These DETs are involved in signalling responses, apoptosis and virus transmission. Protein metabolism is also affected in whiteflies by *Begomovirus* infection, and genes associated with protein synthesis and amino acid metabolism were largely downregulated in virus-infected whiteflies [38,61, 66].

Virus infection can induce DNA damage, and in response, host cells activate DNA repair mechanisms [69]. In our study, upregulation of a heterochromatin protein 1-like isoform X1 was identified, which is a major component of heterochromatin and known for its diverse nuclear functions in transcriptional regulation and nuclear architecture [70]. Pumilio homolog 3, an inhibitor of DNA damage sensor, named Poly (ADP-ribose) polymerase-1 (PARP1) was also upregulated. PARP1 acts as a first responder to DNA damage and initiates DNA repair signalling [71]. Downregulation of pumilio homolog 3 may result in increased activity of PARP1, promoting DNA repair process in nucleus. Apoptosis is another response to virus infection and can reduce viral replication, infectivity and spread within the insect host [72,73]. In our study, several DETs involved in the apoptosis pathway were downregulated, which may limit spread of the virus within the vector. Genes involved in DNA repair and apoptosis were also altered in virus-infected whiteflies [38]. Overall, these alterations of the cell functions by viruses may be an adaptation or a defensive mechanism of insect vectors in response to viral infection.

## Conclusion

This study represents the first transcriptomic analysis of BLH in response to BCTV infection, providing novel insights into the molecular mechanisms influencing vector performance and virus-vector interactions. We demonstrated a significant impact of BCTV on BLH performance, with viruliferous insects showing increased nymphal production compared to non-viruliferous counterparts, potentially enhancing virus transmission. Comparative transcriptomic analyses identified global transcriptional pathways modulated in viruliferous BLHs, revealing the upregulation of crucial genes associated with reproduction, immunity and stress, which may limit virus spread in the insect vector. Interestingly, a concomitant downregulation of DETs related to movement and dispersal was observed, suggesting a potential trade-off between reproduction and mobility in viruliferous BLHs. Furthermore, changes in primary metabolism, including upregulation of DETs associated with energy metabolism, namely glucosidases, lipid digestion and transport, and protein degradation, were observed. Lastly, our findings also highlight the intricate modulation of cellular functions by BCTV, including alterations in metabolism, DNA repair mechanisms and apoptosis pathways, likely representing adaptive responses or defensive mechanisms of BLHs against viral infection. Overall, this study identifies key pathway players of virus manipulation of the vector and vector defensive strategies as the result of long-term co-adaptation and co-evolution of begomoviruses and their insect vectors. These findings provide a valuable foundation for identifying novel gene targets for functional genomics studies and contribute to the development of strategies to control BLH vector populations and block the spread of BCTV by disrupting vital gene pathways and virus-vector interactions.

## Supplementary material

10.1099/jgv.0.002012 Uncited Supplementary Material 1.

10.1099/jgv.0.002012 Uncited Supplementary Material 2.

10.1099/jgv.0.002012 Uncited Supplementary Material 3.

10.1099/jgv.0.002012 Uncited Supplementary Material 4.

## Abbreviations

BCTV: beet curly top virus
BLH: beet leafhopper
BP: biological process
BUSCO: Benchmarking Universal Single-Copy Ortholog
CC: cellular component
DET: differentially expressed transcript
FDR: false discovery rate
GO: Gene Ontology
MED: Mediterranean
MF: molecular function
PCA: principal component analysis
RNA-Seq: RNA sequencing
RT-qPCR: reverse transcription-quantitative PCR
ToCV: tomato chlorosis virus
TYLCCNV: tomato yellow leaf curl China virus
TYLCV: tomato yellow leaf curl virus
VST: variance stabilizing transformation
