# Supplementary material for: Beet curly top virus affects vector biology: the first transcriptome analysis of the beet leafhopper
Source: J Gen Virol. 2024 Jul 29;105(7):002012. doi: 10.1099/jgv.0.002012 (PMC12453401; doi:10.1099/jgv.0.002012)
Supplement: Uncited Supplementary Material 1. [file jgv-105-02012-s001.pdf]

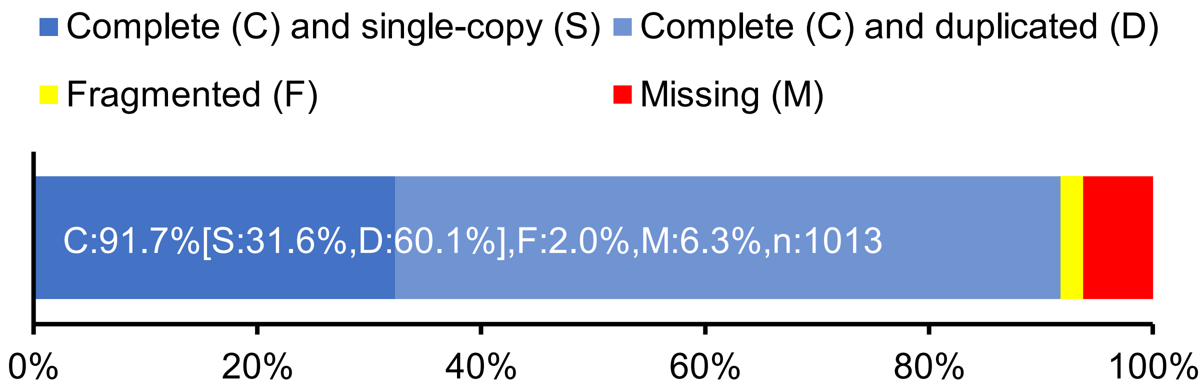

Figure S1. Quantitative assessment of the completeness of the assembled beet leafhopper transcriptome. The Benchmarking Universal Single-Copy Orthologs (BUSCO) analysis was performed to evaluate the completeness of the assembled transcriptome by comparing to the lineage dataset of Arthropoda_Odb10.

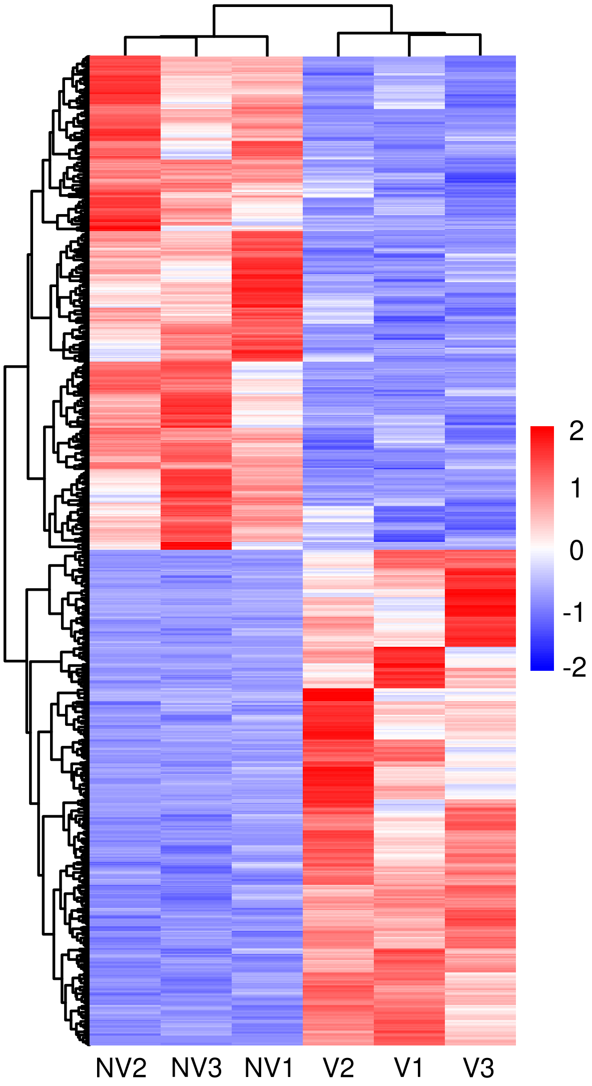

Figure S2. Hierarchical clustering of differentially expressed transcripts across the beet leafhopper samples. Heatmap shows the differential expressions between viruliferous (V) and non-viruliferous (NV) beet leafhoppers. Expression values are Z-score transformed across all samples. Color key indicates higher (red) to lower (blue) expression levels. Rows and columns indicate genes and insect samples respectively.

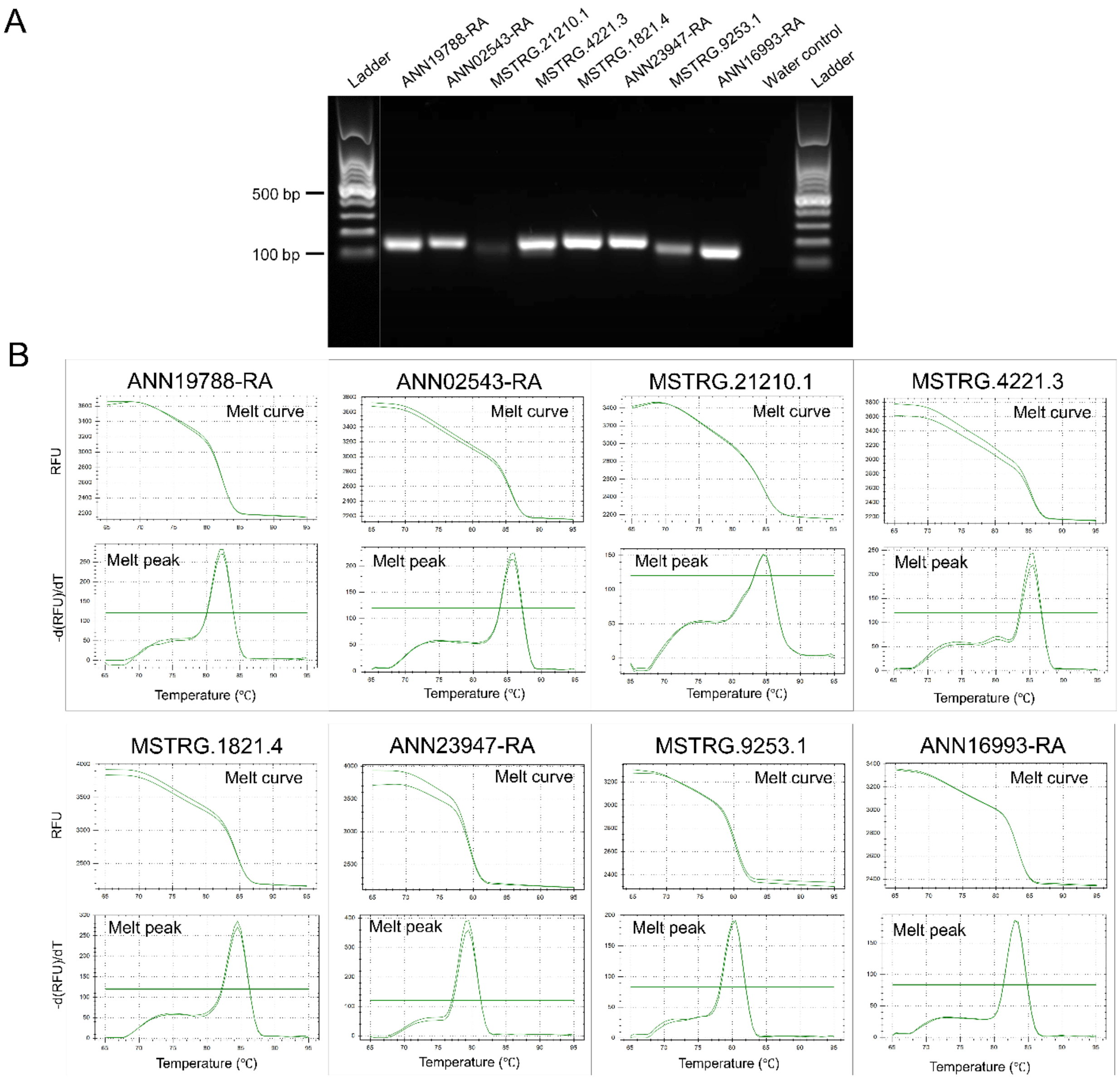

Figure S3. Primer specificities. A) Agarose gel showing the unique PCR amplification band for each primer pair. B) Melting curves and melting peaks for each primer pair. Each green line represents a technical replicate of RT-qPCR reaction.

Table S1. List of primer pairs used for RT-qPCR analysis

Transcript ID	Strand	Sequence (5' to 3')	Length (bp)	Annealing temperature (ºC)	Product size (bp)	Primer efficiency (%)	*Reference
BCTV	Forward	GTGGATCAATTTCCAGACAATTATC	24	58	496	NA	[1]
	Reverse	CCCATAAGAGCCATATCAAACTTC	24	58			
MSTRG.4221.3	Forward	CAATCCTACGAGAACGGGCA	20	60	172	109	This study
	Reverse	TGACGCAGTGGATGTAGTCC	20	60			
MSTRG.21210.1	Forward	ACTGTACTCTACCGCCTCGT	20	60	123	98	This study
	Reverse	TTAGTGGTGGACCAAGCTGC	20	60			
ANN02543-RA	Forward	ACTCTGCGGCTTAATGGAGG	20	60	158	101	This study
	Reverse	TGGGAGATGCAGGTTGACAC	20	60			
ANN19788-RA	Forward	ATGTGAATGTGTGTGCGTGC	20	60	134	102	This study
	Reverse	TAAATGCAGGGGAAGCTCCG	20	60			
ANN16993-RA	Forward	GCTGGTGCTGAGTATGTCGT	20	60	157	94	This study
	Reverse	GGTCGTAGGCATCCAGGTTC	20	60			
MSTRG.9253.1	Forward	TCCAGTGACATGGCGTCTTC	20	60	146	106	This study
	Reverse	ACACTGCCCCGACTGAATTT	20	60			
ANN23947-RA	Forward	GCGTTGATGACACCGTCCTA	20	60	194	97	This study
	Reverse	GTCCTTCTTCCTGACGGCAA	20	60			
MSTRG.1821.4	Forward	CATCAGGATCAAGCCCCTGG	20	60	189	100	This study
	Reverse	GCCTTGTGCCTGAGTTGTTG	20	60			

Actin	Forward	GAGAAGTCCTACGAGCTGCC	20	60	191	97	This study
	Reverse	GACAGGACAGTGTTGGCGTA	20	60			
Ribosomal protein L13	Forward	TCAAGACTGTGGTGATGCCC	20	60	104	105	This study
	Reverse	CTTCCTGAGGGCTGTGAAGG	20	61			

*1. Strausbaugh CA, Eujayl IA, Wintermantel WM. Beet curly top virus strains associated with sugar beet in Idaho, Oregon, and a Western US Collection. *Plant disease*. 2017;101(8):1373-82.

Table S2. Statistic summary of RNA-Seq reads for non-viruliferous (NV) and viruliferous (V) beet leafhoppers from three biological replications

Sample	NV1	NV2	NV3	V1	V2	V3	Mean
Total raw reads (bp)	7,173,518,948	6,124,295,750	3,736,764,988	4,570,729,532	6,327,809,020	5,457,906,476	4,426,782,798
Total filtered reads (bp)	6,038,186,630	4,724,437,296	2,531,132,175	3,592,289,101	5,234,193,549	4,440,458,034	4,277,408,167
Average length of filtered reads (bp)	147	147	147	147	147	147	147
*Total filtered Q20 reads (%)	97	97	97	97	97	96	97
#Total filtered Q30 reads (%)	91	91	91	91	91	90	91

*Percentage of reads with no less than Phred score (Q) of 20; # Percentage of reads with no less than Phred score (Q) of 30.

Table S3. Differentially expressed transcripts identified in the beet leafhopper in response to beet curly top virus infection

This file is provided in a separate excel file.

Table S4. Raw RT-qPCR Cq values for eight target transcripts in viruliferous (V) and non-viruliferous (NV) beet leafhoppers

This file is provided in a separate excel file.

Table S5. Statistical analysis of normalized expression of eight target transcripts between viruliferous (V) and non-viruliferous (NV) beet leafhoppers

This file is provided in a separate excel file.
